# Supplementary material for: Phytochemical-rich medicinal plant extracts suppress bacterial antigens-induced inflammation in human tonsil epithelial cells
Source: PeerJ. 2017 Jun 22;5:e3469. doi: 10.7717/peerj.3469 (PMC5483044; doi:10.7717/peerj.3469)
Supplement: Supplemental Information 1 [file peerj-05-3469-s001.docx]

**Table S1: Effect of ethanol extracts on percentage cell viability of human tonsil epithelial cells.**

| Test compounds | Cell viability (%) | | | | | | | |
| --- | --- | --- | --- | --- | --- | --- | --- | --- |
|  | **0.1 μg/mL** | **0.5 μg/mL** | **1 μg/mL** | **5 μg/mL** | **10 μg/mL** | **25 μg/mL** | **50 μg/mL** | **100 μg/mL** |
| PGN+LTA | 96.8±1.1^ab^ | ND | ND | ND | ND | ND | ND | ND |
| Licorice R | 99.0±1.6^a^ | 99.1±1.6^a^ | 98.0±1.7^a^ | 98.4±1.7^a^ | 97.1±1.7^a^ | 32.6±1.3^ef^ | 24.4±0.7^f^ | 22.5±1.2^fg^ |
| Sage L | 99.2±1.2^a^ | 99.8±0.4^a^ | 96.2±6.0^a^ | 86.2±1.1^c^ | 33.1±1.7^a^ | 29.5±0.8^ef^ | 24.4±0.9^f^ | 25.3±1.8^f^ |
| Echinacea S | 82.8±1.3^c^ | 82.8±3.7^c^ | 95.8±3.7^a^ | 83.0±2.1^c^ | 52.5±2.6^d^ | 26.2±1.3^ef^ | 20.9±0.6^g^ | 20.4±1.0^g^ |
| Echinacea F | 97.0±0.0^a^ | 97.1±2.0^a^ | 96.1±3.1^a^ | 97.1±1.1^a^ | 97.5±5.5^a^ | 27.5±0.1^ef^ | 18.2±1.3^g^ | 18.2±0.6^g^ |
| Oregano FS | 96.9±1.5^a^ | 96.9±1.9^a^ | 96.9±1.0^a^ | 86.9±1.0^c^ | 85.3±0.8^c^ | 68.4±0.1^d^ | 67.7±3.2^a^ | 25.2±2.0^f^ |
| Theme FS | 95.6±3.2^a^ | 95.6±3.2^ab^ | 96.6±3.2^ab^ | 95.6±2.1^a^ | 92.5±0.8^b^ | 90.4±1.9^b^ | 82.8±2.8^c^ | 78.9±0.6^c^ |
| Barberry R | 97.5±0.5^a^ | 97.5±0.7^a^ | 95.5±0.4^ab^ | 97.5±1.0^a^ | 97.7±0.4^a^ | 92.8±0.4^b^ | 98.2±0.2^a^ | 87.8±1.5^bc^ |
| Slippery elm IB | 95.2±1.5^ab^ | 93.4±1.1^b^ | 89.7±1.0^b^ | 82.7±1.0^c^ | 76.0±3.0^c^ | 23.9±0.4^fg^ | 20.7±1.2^g^ | 21.0±1.7^g^ |
| Clove FB | 96.1±1.1^a^ | 97.5±1.9^a^ | 96.5±1.9^ab^ | 97.5±1.3^a^ | 98.7±3.2^a^ | 73.0±8.4^c^ | 36.0±1.6^e^ | 34.7±2.5^e^ |
| Ginger Rh | 98.5±1.1^a^ | 98.9±0.6^a^ | 98.9±1.6^a^ | 98.9±1.2^a^ | 98.8±5.3^a^ | 91.4±2.4^b^ | 96.4±1.6^a^ | 40.1±1.0^e^ |
| Olive L | 99.8±1.3^a^ | 96.8±1.6^ab^ | 98.8±1.7^a^ | 98.8±2.0^a^ | 95.4±7.2^a^ | 94.4±5.8^ab^ | 20.7±1.2^g^ | 21.0±1.7^g^ |
| Geranium L | 95.2±1.3^ab^ | 85.1±2.0^c^ | 46.1±2.0^de^ | 41.1±0.0^e^ | 36.8±0.8 ^a^ | 28.0±1.9^ef^ | 31.8±2.7^a^ | 21.4±2.0^g^ |
| Echinacea L | 97.9±1.1^a^ | 97.3±1.4^a^ | 97.9±1.5^a^ | 97.9±0.6^a^ | 88.0±2.0^b^ | ND | 42.0±1.6^a^ | 28.1±1.5^ef^ |
| Danshen R | 98.2±1.0^a^ | 95.9±1.1^ab^ | 98.9±1.0^a^ | 98.9±1.1^a^ | 65.3±1.5^d^ | ND | 26.2±2.7^f^ | 18.8±1.4^g^ |
| Nemesulide | 97.1±1.2^a^ | 97.4±1.0^a^ | 95.8±1.8^ab^ | 95.8±1.8^ab^ | 95.7±4.5^a^ | 96.6±5.0^a^ | 89.9±2.1^b^ | 86.0±2.3^bc^ |

**Cells were treated with various concentration of test compounds for 24 hr. Cell viability (%) was calculated relative to the control of 0.05% DMSO. Values of the same column are expressed as mean±SD (n=3), Tukey’s test (p≤ 0.05). The value with different letters indicating the significant difference. ND: not determined; F: flowers; FB: flower bud; FS: flowering shoots; IB: inner bark; L: leaves; Rh: rhizome; R: roots; S: stem.**

| Test compounds | Cell viability (%) | | | | | | | |  |
| --- | --- | --- | --- | --- | --- | --- | --- | --- | --- |
|  | **0.1 μg/mL** | **0.5 μg/mL** | **1 μg/mL** | **5 μg/mL** | **10 μg/mL** | **25 μg/mL** | **50 μg/mL** | **100 μg/mL** | |
| PGN+LTA | 96.4±1.6^ab^ | ND | ND | ND | ND | ND | ND | ND | |
| Licorice R | 98.7±1.0^a^ | 96.4±1.6^a^ | 97.1±1.0^a^ | 80.7±1.8^c^ | 95.0±3.5^ab^ | 97.5±1.0^a^ | 97.7±1.9^a^ | 98.2±1.3^a^ | |
| Oregano FS | 97.8±1.1^a^ | 98.7±1.1^a^ | 98.7±1.0^a^ | 89.4±5.1^b^ | 90.2±1.9^b^ | 88.1±3.8^b^ | 97.8±2.0^a^ | 25.2±4.0^e^ | |
| Thyme FS | 98.2±0.5^a^ | 97.8±1.1^a^ | 98.0±1.0^a^ | 84.2±3.6^c^ | 93.2±4.2^ab^ | 96.4±2.9 ^ab^ | 96.6±1.0^a^ | 94.1±0.4^b^ | |
| Barberry R | 99.3±2.0^a^ | 98.2±0.5^a^ | 98.1±0.0^a^ | 99.1±3.2^a^ | 99.6±0.3^a^ | 95.3±0.7^a^ | 99.2±3.5^a^ | 96.4±3.8^a^ | |
| Echinacea S | 99.1±1.1^a^ | 99.3±2.0^a^ | 97.1±0.1^a^ | 97.4±1.0^a^ | 96.3±0.5^a^ | 91.5±1.3^b^ | 95.5±2.7^a^ | 58.3±5.3^d^ | |
| Echinacea F | 98.1±1.1^a^ | 99.1±1.1^a^ | 96.1±1.1^a^ | 98.9±0.9^a^ | 95.6±4.5^a^ | 91.2±1.3^b^ | 97.2±2.9^a^ | 97.3±3.3^a^ | |
| Echinacea L | 99.1±1.5^a^ | 98.1±1.1^a^ | 97.0±1.2^a^ | 96.0±0.6^a^ | ND | ND | 99.7±1.9^a^ | 99.6±3.9^a^ | |
| Sage L | 97.1±0.8^a^ | 99.0±1.5^a^ | 97.0±1.1^a^ | 97.6±1.9^a^ | ND | ND | 103.5±0.9^a^ | 99.7±1.9^a^ | |
| Clove FB | 98.5±1.7^a^ | 98.4±0.0^a^ | 98.9±0.3^a^ | 98.3±1.1^a^ | ND | ND | 92.7±3.4^b^ | 87.3±4.3^b^ | |
| Ginger Rh | 99.0±1.1^a^ | 98.1±0.1^a^ | 98.4±1.0^a^ | 98.7±0.5^a^ | ND | ND | 98.6±4.8^a^ | 98.3±2.8^a^ | |
| Olive L | 97.2±1.0^a^ | 97.0±1.7^a^ | 96.0±1.6^a^ | 96.2±1.0^a^ | ND | ND | 99.7±1.9^a^ | 94.6±2.4^b^ | |
| Geranium L | 97.1±0.2^a^ | 98.2±1.2^a^ | 99.0±1.1^a^ | 93.1±1.0^ab^ | ND | ND | 45.7±6.1^d^ | 25.2±1.5^e^ | |
| Slippery elm IB | 99.1±1.3^a^ | 98.0±1.5^a^ | 96.3±1.7^a^ | 97.1±1.3^a^ | ND | ND | 99.7±4.8^a^ | 99.6±2.4^a^ | |
| Nemesulide | 99.4±1.0^a^ | 98.0±1.6^a^ | 98.1±1.4^a^ | 96.0±1.1^a^ | 95.7±4.5^a^ | 96.6±3.0^ab^ | 89.9±2.1^b^ | 86.0±3.0^bc^ | |

**Table S2: Effect of aqueous extracts on percentage cell viability of human tonsil epithelial cells.**

107

**Cells were treated with various concentration of test compounds for 24 hr. Cell viability (%) was calculated with relative to the control of 0.05% DMSO. Values are expressed as mean±SD (n=3), Tukey’s test (p≤ 0.05). The value with different letters indicating the significant difference. ND: not determined; F: flowers; FB: flower bud; FS: flowering shoots; IB: inner bark; L: leaves; Rh: rhizome; R: roots; S: stem.**
